# C57BL/6J mice exposed to perfluorooctanoic acid demonstrate altered immune responses and increased seizures after Theiler’s murine encephalomyelitis virus infection

**DOI:** 10.3389/fimmu.2023.1228509

**Published:** 2023-08-02

**Authors:** Aracely A. Pérez Gómez, Meichen Wang, Kelli Kochan, Katia Amstalden, Colin R. Young, C. Jane Welsh, Timothy D. Phillips, Candice L. Brinkmeyer-Langford

**Affiliations:** ^1^Interdisciplinary Faculty of Toxicology, School of Veterinary Medicine and Biomedical Sciences, Texas A&M University, College Station, TX, United States; ^2^Department of Veterinary Integrative Biosciences, School of Veterinary Medicine and Biomedical Sciences, Texas A&M University, College Station, TX, United States; ^3^Texas A&M Institute for Genome Sciences and Society, Texas A&M University, College Station, TX, United States

**Keywords:** TMEV, PFOA, cytokines, chemokine, seizures

## Abstract

**Introduction:**

Neurological diseases can stem from environmental influences such as antecedent viral infections or exposure to potential toxicants, some of which can trigger immune responses leading to neurological symptoms. Theiler’s murine encephalomyelitis virus (TMEV) is used to model human neurological conditions associated with prior viral infections, with outcomes partly attributable to improper induction and regulation of the immune response. Perfluorooctanoic acid (PFOA) can alter pathologies known to influence neurological disease such as inflammatory responses, cytokine expression, and glial activation. Co-exposure to TMEV and PFOA was used to test the hypothesis that early life exposure to the potential immunotoxicant PFOA would affect immune responses so as to render TMEV-resistant C57BL/6J (B6) mice susceptible to viral-induced neurological disease.

**Methods:**

Neonate B6 mice were exposed to different treatments: non-injected, sham-infected with PBS, and TMEV-infected, with the drinking water of each group including either 70 ppt PFOA or filtered water. The effects of PFOA were evaluated by comparing neurological symptoms and changes in immune-related cytokine and chemokine production induced by viral infection. Immune responses of 23 cytokines and chemokines were measured before and after infection to determine the effects of PFOA exposure on immune response.

**Results:**

Prior to infection, an imbalance between Th1, Th2, and Treg cytokines was observed in PFOA-exposed mice, suppressing IL-4 and IL-13 production. However, the balance was restored and characterized by an increase in pro-inflammatory cytokines in the non-infected group, and a decrease in IL-10 in the PFOA + TMEV group. Furthermore, the PFOA + TMEV group experienced an increase in seizure frequency and severity.

**Discussion:**

Overall, these findings provide insight into the complex roles of immune responses in the pathogenesis of virus-associated neurological diseases influenced by co-exposures to viruses and immunotoxic compounds.

## 1 Introduction

Per-and polyfluoroalkyl substances (PFAS) are a group of compounds globally distributed in the environment because of their amphipathic properties and exceptional stability against chemical and thermal degradation (1, 2). Over the years, PFAS have been used in commercial and industrial applications for fire prevention, insulation, non-stick coatings, medical and personal care products, textiles, packaging, and many more (3, 4). Two main compounds, perfluorooctane sulfonate (PFOS) and perfluorooctanoic acid (PFOA), are considered as “legacy PFAS” which form the structural basis for the creation of new generation (GenX) PFAS compounds (5). While PFOS and PFOA are no longer manufactured, both legacy compounds remain prevalent in drinking water and their bioaccumulation is evident in >98% of serum samples taken from the general US population (6, 7).

PFAS exposure has been linked to adverse effects, such as potentially altering development, lipid metabolism, and the endocrine system (8–10). Despite the presence of a specific mode of action, PFAS can also have carcinogenic, endocrine, hepatotoxic, and immunotoxic effects (11–14). For example, PFOA exposure results in suppression of antibody responses, increased hypersensitivity-related effects, alteration of T cell proliferation, and diminished cytokine release (15–17). Consequently, PFAS exposure influences the effectiveness of immunizations and increases symptom susceptibility after viral infection (18, 19), and PFAS have been classified as potential immune hazards by the National Toxicology Program (NTP) (20).

PFAS such as PFOS and PFOA may cross the blood-brain barrier (BBB) and exert neurological effects (21, 22). Exposure to PFOA can reduce synapse densities and alter the structure of the hippocampus (23). High doses of PFOA may also diminish myelin content in the brain and during neurogenesis (24). Furthermore, PFOA was found to impair neurodevelopmental processes mainly due to the pathological suppression of normal immune responses (25, 26). Overall, PFAS exposure can interfere with motor function, myelination, microglial activation, and pro-inflammatory cytokine expression; thus, PFAS has the potential to alter immunological and neurological processes (14, 27).

Not only can PFAS exposure alter immunological and neurological processes, but neurotropic viral infections have also been associated with neurological diseases. While PFAS exposure can interfere with immune function, leading to immune suppression or dysregulation, viral infections typically trigger an immune response aimed at eliminating the virus which in some cases can contribute to pathological damage. Infection with a single virus is known to evoke a spectrum of immunological outcomes depending on the host’s genetic background (28, 29). Previous studies have linked neurotropic viral infections, such as Epstein-Barr virus (EBV), Herpes Simplex virus (HSV), human immunodeficiency virus (HIV), poliovirus, Zika virus, and more recently, COVID-19, to pathologies resulting in neurological diseases (e.g., amyotrophic lateral sclerosis [ALS], epilepsy, multiple sclerosis [MS], and Parkinson’s disease [PD]) (30–34).

Mice infected by Theiler’s murine encephalomyelitis virus (TMEV), a naturally occurring neurotropic single-stranded RNA murine virus, have historically been used to model human neurological symptoms associated with viral infections (35–37). TMEV infection has furthermore been used to model a broad array of neurological sequelae influenced by the genetic background of the infected mouse strain. Based on these responses, mouse strains have been classified as TMEV-susceptible, -resistant, or -resilient (38, 39). TMEV-resistant strains (e.g., C57BL/6J [B6]) clear the virus within two to three weeks post-infection, while TMEV infection persists in resilient and susceptible strains, with susceptible strains showing greater clinical severity than resilient strains. The balance between pro-inflammatory and anti-inflammatory cytokines plays a crucial role in determining susceptibility. In particular, the production of interferon-gamma (IFN-γ) and interleukin-10 (IL-10) has been implicated in modulating TMEV susceptibility, with high levels of IFN-γ associated with resistance and elevated IL-10 levels associated with susceptibility. Furthermore, improper induction and regulation of immune responses to TMEV can affect neurological responses to TMEV infection. However, it is crucial to assess how other environmental factors may synergistically contribute to these outcomes.

Co-exposure to both PFOA and TMEV was hypothesized to result in suppressed cytokine and chemokine production following TMEV infection, leading to viral persistence and increased neurological symptoms in TMEV-resistant B6 mice. To test this hypothesis, the potential synergistic immune effects of PFOA and TMEV exposure were evaluated using the PFOA dose of 70 ppt based on typical human exposure and EPA’s regulatory measures for drinking water limits (40). **TMEV-resistant B6 mice typically exhibit seizures** within the first few days post infection, prior to clearing the virus (41, 42); therefore, behavioral symptoms were evaluated for 14 days post-infection (dpi) and TMEV RNA levels were quantified in the hippocampus and spinal cord. Cytokine and chemokine profiles were characterized in serum samples collected before and after TMEV infection to identify associations between immune responses, neurobehavioral effects, and viral transcript levels. The observations presented in this study add to the growing body of evidence of the adverse effects of PFOA, specifically its immunotoxic effects. The results provide significant information on PFAS-related immune alterations and highlight their impact on neurological disease after a viral infection.

## 2 Materials and methods

### 2.1 Ethics statement

All animal care protocols were approved by Texas A&M University Laboratory Animal Care and Use Committee (AUP 2020-0065, approved May 21, 2020) and complied with NIH Guidelines for Care and Use of Laboratory Animals. Mice were group-housed under controlled standard conditions in an ambient temperature room and 12-hour light/dark cycles, with ad libitum food and water. All testing was performed during the light phase.

### 2.2 Animal and exposure treatments

Female and male C57BL/6J mice were allowed to mate and deliver pups without intervention. Once pregnant, female mice were checked every morning for parturition. Day of birth was designated as post-natal day (PND) 0. Litters contained pups of both sexes during the neonatal period and no sex-based separation was made until weaning at PND 21. The quantity of mice in this study depended on the reproductive success of our in-house breeding program.

**Figure 1:**
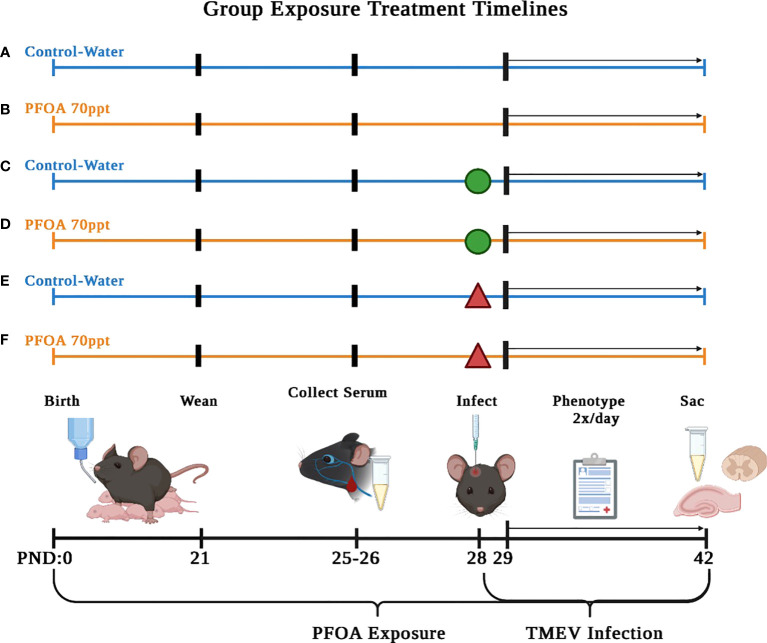
Serum collected on PND 42 (14 dpi) was used to compare cytokine and chemokine levels to identify PFOA-induced. The six treatment groups described in this study are as follows: **(A)** no injection of mice and no PFOA exposure, **(B)** no injection of mice and 70 ppt PFOA exposure, **(C)** sham-infected mice and no PFOA exposure, **(D)** sham-infected mice and 70 ppt PFOA exposure, **(E)** TMEV-infected mice and no PFOA exposure, and **(F)** TMEV-infected mice and 70 ppt PFOA exposure. Figure created with BioRender.com.

On PND 0, full litters were randomly sorted (**Table 1**) yielding the following six exposure groups (**Figure 1**): (A) PFOA control + No injection– pups were supplied with deionized drinking water and did not undergo TMEV injection procedure; (B) PFOA + No injection– pups were supplied with 70 ppt PFOA *via* drinking water and did not undergo TMEV injection procedure; (C) PFOA control + Sham-injected– pups were supplied with deionized drinking water and were injected with Phosphate Buffer Solution (PBS), as described below in “Virus Inoculation”; (D) PFOA + Sham-injected– pups were supplied with 70 ppt PFOA *via* drinking water and were injected with PBS; (E) PFOA control + TMEV-infected– pups were supplied with deionized drinking water and were injected with TMEV; (F) PFOA + TMEV-infection– pups were supplied with 70 ppt PFOA *via* drinking water and were injected with TMEV.

**Table 1:** C57BL/6J mice were allocated to treatment groups after birth (see **Figure 1** for timelines).

Group	PFOA (ng/mL)	TMEV	Sex	*n*
A	0	No*	Female	4
	0	No*	Male	4
B	70	No*	Female	4
	70	No*	Male	4
C	0	S	Female	6
	0	S	Male	7
D	70	S	Female	4
	70	S	Male	4
E	0	I	Female	6
	0	I	Male	7
F	70	I	Female	4
	70	I	Male	4

* indicates mice were not injected with either PBS or TMEV. S indicates sham-infected with PBS; I indicates infected with TMEV.

### 2.3 Dosing solutions

Crystallized PFOA (96% purity; Millipore Sigma Chemical Company, St. Louis, MO, USA) was dissolved by agitation in deionized water at a stock concentration of 0.07 mg/mL. PFOA-dosed drinking water was prepared by diluting the PFOA stock to a final concentration of 70 ng/L (ppt), a human relevant dose and EPA’s standard for PFOA in drinking water [41,96]. PFOA drinking water was prepared fresh bi-weekly and water bottles were refilled weekly. Mice in PFOA exposed groups were given 70 ppt PFOA in drinking water (Groups B, D, and F) or deionized water (PFOA control) (Groups A, C, and E) starting at PND 1 until the end of the study (PND 42). Pups were exposed *via* milk from the dams until weaning at PND 21. Mice have been shown to exhibit significant developmental effects following PFOA exposure from either drinking water or from milk (3, 43). Water consumption was measured every morning to ensure PFOA intake.

### 2.4 Virus inoculation

At PND 28, mice in Groups C, D, E, and F were anesthetized by isoflurane inhalation (MWI, Meridian, ID, USA) and intracerebrally (i.c.) injected into the right mid-parietal cortex at a depth of ~1.5 mm with 20 µL of 1 × PBS (Groups C and D) or with 5.0 x 104 plaque-forming units (PFU) of TMEV BeAn strain (Groups E and F; American Type Culture Collection [ATCC] VR 995, Manassas, VA, USA), as previously described (29, 44–46).

### 2.5 Qualitative neurological phenotyping

All mice were weighed and evaluated for TMEV-induced clinical signs (phenotypes) from PND 28 to 42. Phenotypes such as kyphosis, piloerection, limb clasping, and delay in righting reflex were evaluated and scored as described previously (46). Clinical signs of limb weakness and paralysis were observed and scored on a scale of 0 – 4, with a score of 0 given to mice having normal stride and no signs of weakness, and a score of 4 representing the total loss of limb mobility characterized by lack of grip function and flaccid limb extension (45). Seizures were scored between 0 – 5, based on the Racine scale, with a score of 0 given to mice having no seizures, up to a score of 5 given to mice experiencing generalized tonic-clonic seizures accompanied by falling on back and rearing (29).

### 2.6 Serum collection and necropsy

At PND 25 – 26, blood was collected from the submandibular vein *via* puncture with a lancet at a depth of ~1 mm. At the end of study (PND 42), mice were euthanized by intraperitoneal (IP) injection of Beuthansia at 150 mg/kg (Merck & Co., Kenilworth, NJ, USA) as described previously (45, 47, 48). Thereafter, blood was collected from the right atrium and was refrigerated at 4°C for an hour. Blood was then centrifuged at 2,000 × rpm to collect sera and was then stored at –20°C for further analysis. Following blood collection, mice were perfused with a 1 × PBS solution through the left ventricle. Then, thoracic spinal cord and hippocampus were removed and flash-frozen in liquid nitrogen. These tissues were selected for analysis based on their known involvement in TMEV infection (49–51). Tissues were stored at -80°C for RNA extractions, as described below.

### 2.7 Cytokine and chemokine assays

Cytokine and chemokine levels were measured in the sera collected at PND 25 – 26 and at PND 42. Sera collected at PND 25 – 26 was considered as the baseline timepoint when determining TMEV related responses and for comparing with levels observed at the end of the study (PND 42; 14 dpi). Immune response proteins were measured with Bio-Plex Pro™ Mouse Cytokine 23-plex Assay kit (Bio-Rad, Hercules, CA, USA) to determine concentration levels of 23 cytokines and chemokines (IL-1α, IL-1β, IL-2, IL-3, IL-4, IL-5, IL-6, IL-9, IL-10, IL-12 [p40], IL-12 [p70], IL-13, IL-17α, IFN-γ, CCL11 [Eotaxin], G-CSF, GM-CSF, CXCL1 [KC keratinocyte-derived chemokine], CCL2 [MCP-1 Monocyte Chemotactic Peptide 1]), CCL3 [MIP-1α Macrophage Inflammatory Protein1α], CCL4 [MIP-1β], CCL5 [RANTES], and TNF-α). Data was processed using the Bio-Plex Manager software program (Bio-Rad version 4.1.1, Hercules, CA, USA).

### 2.8 RNA isolation and TMEV RNA detection

Thoracic spinal cord and hippocampal samples were thawed at room temperature, and RNA extractions were performed using Maxwell-16^®^ automated equipment with a LEV Simply RNA tissue kit (Promega, Sunnyvale, CA, USA). RNA quality and concentration was verified using an Agilent Tape Station with all samples having a RIN score over 8.0 (Agilent Technologies, Santa Clara, CA, USA).

To detect TMEV RNA in both CNS tissues, 125 ng of total RNA was reverse-transcribed to cDNA in 20 µL reactions using Superscript II (ThermoFisher Scientific, Waltham, MA, USA) with gene-specific primers at a final concentration of 10 µM. The resulting cDNA was diluted (1:5) in nuclease-free water.

Primers were designed using the Oligo 7 Primer Analysis Software version 7.6 (Molecular Biology Insights, Cascade, CO, USA). Primers for TMEV were designed from the BeAn strain sequence (M16020.1): Forward 5’–CTG CAA ACA TGG ATA CCC AGA T–3’; Reverse 5’–GTC CAC ACA AAG AAG GTC CGT A–3’; Standard oligonucleotide length: 131 nucleotides (Integrated DNA Technologies, Coralville, IA, USA). Primer pairs were assessed *via* a BLAST sequence similarity search to avoid cross-amplification across different species and organ systems or between different mRNA transcripts (52). Real-time quantitative PCR (RTq-PCR) was performed according to standard protocols. The sample order was randomized, and amplifications were repeated in triplicate in a 384-well plate at a total reaction volume of 20 µL, containing 10 µL Power SYBR Green PCR Master Mix (ThermoFisher Scientific, Waltham, MA, USA), 0.6 µL of each primer pair at 10 µM, 6.8 µL nuclease-free water, and 2.0 µL of template cDNA. A standard curve was constructed using the linear range of the 10‐fold serial dilution standards ranging from 1012 to 103 copies/µL for absolute quantification. PCR reactions were run on a CFX-384 Touch Real-Time PCR Detection System (Bio-Rad, Hercules, CA, USA) and analyzed using Bio-Rad CFX Maestro Software (version 1.1).

### 2.9 Chemical *a*nalysis

Serum samples of 160 µL were thawed for 1 min in a water bath (37°C) and mixed with 640 μL of 50% acetonitrile containing 0.2% formic acid. After centrifugation at 8000 g for 8 min, a 700 μL aliquot of the supernatant was loaded into Strata-X-AW solid phase extraction (SPE) cartridges (Phenomenex, Torrance, CA, USA) that were preconditioned with methanol and water (53). The loaded SPE cartridges were washed with 1 mL of 25 mM ammonium acetate to eliminate interferences. The analytes were eluted using 1 mL of 5% ammonium hydroxide in methanol, dried in a vacuum evaporator, and reconstituted in 5% ammonium hydroxide in 60% acetonitrile/40% methanol containing 0.05 µM of ^13^C_4_-PFOA (Wellington Laboratory, Canada).

PFAS was analyzed using a Waters Acquity ultraperformance liquid chromatograph/tandem mass spectrometer (LC/MS-MS) equipped with triple quadrupole, following a previous method (54). An Acquity BEH C18 column (2.1 × 50 mm, 1.7 μm) with a VanGuard pre-column (2.1 × 5 mm, 1.7 μm) at 40°C was used for separation. 20 mM ammonium acetate (eluent A) and acetonitrile (eluent B) were carried out at 0.6 mL/min flow rate. The gradient program for elution was 10% eluent B (initial), 10%-55% (0 to 0.1 min), 55%-99% (0.1 to 4.5 min), 99% (4.5 to 5 min), and 99%-10% (5 to 6.5 min). The injection volume was 50 μL for each analysis. The mass spectrometer was operated in a negative ion mode at 4.5 kV spray voltage and 450°C source temperature. The monitored precursor and product ions (m/z) for PFOA and ^13^C_4_-PFOA were 413 to 369, and 417 to 372, respectively, at 20 kV cone voltage. To ensure consistency of the detection methods and linearity of peak concentrations, PFOA standard solutions at concentrations between 0.05 and 50 ng/mL in distilled water were prepared and run with each analysis. The 7-point standard curve was linear (r^2^ > 0.99) with a limit of detection at 0.05 ng/mL and a recovery percentage averaged 114%.

### 2.10 Statistics

GraphPad Prism version 9.5.0 for Mac (GraphPad Software, San Diego, CA, USA) was used to perform one-way analysis of variance (ANOVA) with Tukey’s multiple comparisons test to compare individual phenotypic responses between the six exposure groups. A two-way ANOVA test with Tukey’s multiple comparisons test was used to compare daily weights per exposure group, address sex-specific differences among seizure counts, evaluate TMEV RNA expression in spinal cord tissue, identify sex-specific differences in TMEV RNA expression in CNS tissue, and detect sex-specific differences when comparing 0 ppt and 70 ppt pre-infection cytokine levels. Sidak’s multiple comparison test was implemented to identify sex-specific differences for individual phenotypes, compare seizure counts between the six exposure groups, evaluate TMEV RNA expression in hippocampal tissue, and evaluate serum cytokine levels at PND 42 for PFOA-induced changes and sex-specific differences. To evaluate the influence PFOA on pre-infection cytokine levels among the six exposure cohorts an unpaired t-test was utilized. A Pearson R correlation analysis between serum cytokine levels in Groups E and F and their TMEV RNA expression and seizure frequencies indicated the influence of PFOA on serum cytokine levels in TMEV-exposed groups. All reported p-values were based on two-tailed statistical tests, with a significance level of 0.05.

## 3 Results

### 3.1 The pervasiveness of PFOA in serum was confirmed *via* mass spectrometry

Serum collected at postnatal day (PND) 26 (before infection) and at PND 42 (end of study) was used to measure PFOA concentration levels. PFOA was measured in mice from Groups A (0 ppt + no injection) and B (70 ppt + no injection), but did not statistically differ among the two. In Group B, the average PFOA concentration on day 42 (1.60 ng/mL) was half of the level on day 26 (3.53 ng/mL), correlating with its half-life in serum (55). Low levels of PFOA were also detected in the double blank, single blank, and serum blank samples after 26 and 42 days, showing background contamination and prevalence of PFOA. While serum levels do not necessarily reflect PFOA accumulation in other organs such as the brain, these measurements confirmed the presence of PFOA in the body. Overall, we determined that a low dose of 70 ppt PFOA *via* drinking water can be detected in the serum *via* mass spectrometry (**Supplementary Figure 1**).

### 3.2 Greater weight gain was observed in PFOA-exposed mice, regardless of infection status

Once the mice were weaned and assigned to their respective exposure cohorts, their weights were measured daily from PND 28 (0 days post-infection; dpi) to 42 (14 dpi, the end of the acute phase of TMEV infection). To allow for direct comparisons of weight changes across the different exposure groups, weights were normalized by computing the ratios between the daily weights to the baseline weight measured at 0 dpi (**Supplementary Figure 2A**). Overall, B6 mice exposed to 70 ppt PFOA and without TMEV infection (Groups B and D) displayed the greatest weight gain. TMEV-infected mice exposed to 70 ppt PFOA (Groups E and F) lost weight after infection and regained their original weight by 3 dpi. Thereafter, weights of TMEV-infected mice increased slightly (1.2 × from the original weight). Finally, mice exposed to 70 ppt PFOA and no TMEV infection (Groups C and E) gained the least amount of weight, increasing their starting weight by 1.1 × from their original weight. Weights at 14 dpi were analyzed using two-way ANOVA to determine the differences between the exposure groups’ final weight changes (**Supplementary Table 1**). Weights of mice exposed to 70 ppt PFOA but not infected (Groups B and D) were significantly higher than those of mice exposed to 0 ppt PFOA, regardless of infection status (Groups A, C, and E). These differences and their significance were as follows: 0 ppt + no injection vs. 70 ppt + no injection or 70 ppt + sham-infected, both p < 0.0001; 0 ppt + Sham-infected vs. 70 ppt + no injection or 70 ppt + sham-infected, p < 0.001 and p < 0.0001, respectively; and 0 ppt + TMEV-infected vs. 70 ppt + no injection or 70 ppt + sham-infected, p < 0.05 and p < 0.01, respectively. In addition, the weights of mice exposed to 70 ppt PFOA + sham-infected (Group D) were significantly higher than those of mice exposed to 70 ppt + TMEV-infected (Group F), p < 0.05, suggesting that exposure to 70 ppt PFOA resulted in rapid weight gain. However, exposure to both PFOA and TMEV resulted in slower weight gain; thus, the viral infection curbed the weight gain observed in those solely exposed to 70 ppt PFOA.

To determine whether there were sex differences in weight gain, a two-way ANOVA was performed on the exposure groups. By 14 dpi, all male mice, except for those exposed to 0 ppt PFOA + not injected (Group A), had the greatest weight gain compared with their female counterparts, representing typical weight gain behavior (**Supplementary Figure 2B**).

### 3.3 Neurological phenotypes were more prevalent in PFOA + TMEV exposure group

**Figure 2:**
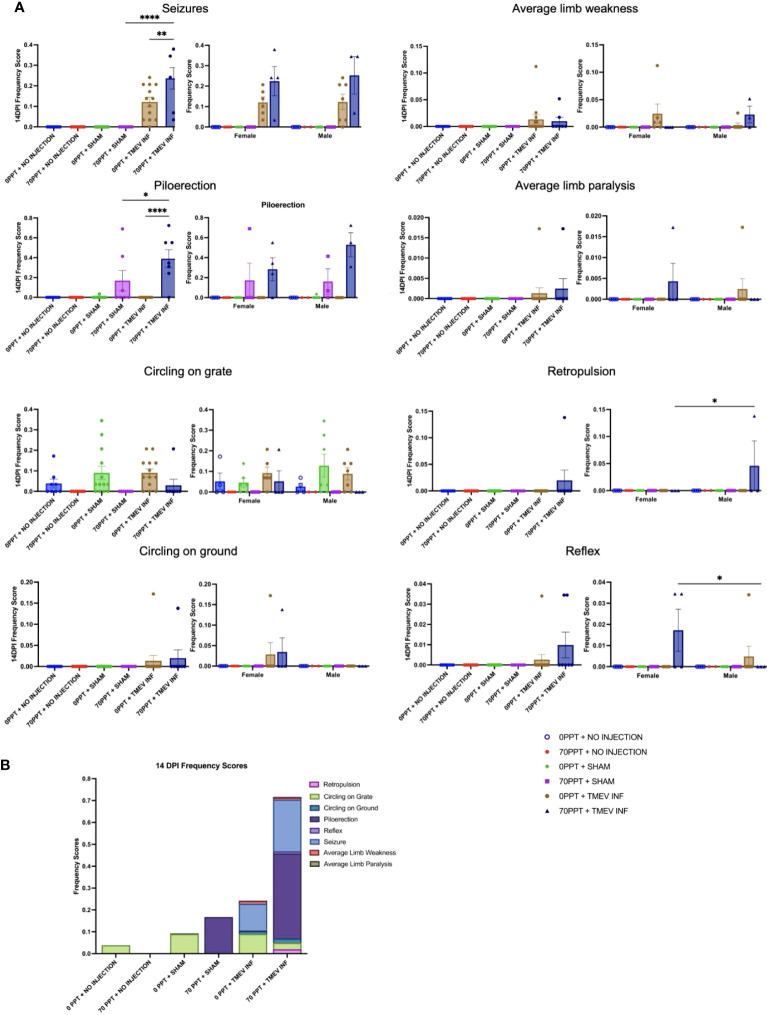
The clinically relevant neurological phenotypes were observed throughout the 14 dpi. **(A)** Frequency scores at 14 dpi were calculated for each phenotype per exposure group (shown on the left for each phenotype). Differences were also compared between females and males, as shown separately on the right. **(B)** Frequency scores were compiled for each phenotype separately, and when considered cumulatively, all observed phenotypes demonstrated the overall clinical response per exposure group. *p < 0.05, **p < 0.01, ****p < 0.0001.

A variety of neurological phenotypes resulting from TMEV exposure have been characterized in different mouse strains (29, 45, 47). B6 mice, considered a TMEV-resistant strain, were used in this study to determine whether PFOA exposure resulted in an increased incidence and/or severity in neurological clinical symptoms. The 14 dpi frequency scores were calculated by dividing the number of observations recorded for each phenotype by the total number of phenotyping events during the acute phase, which included the first 14 days post-infection (**Figure 2A**). All phenotypes were recorded as true observations only when the same phenotypes were not observed in control mice. For example, circling on the grate and ground was observed in multiple treatment groups, regardless of exposure, suggesting circling was a normal behavior for this strain. On the other hand, piloerection, a clinical indicator of sickness, was observed in mice exposed to 70 ppt PFOA and either sham- or TMEV-infected. Piloerection was significantly higher in mice exposed to 70 ppt PFOA + TMEV infection (Group F) than their sham-infected counterparts (Group D; p < 0.05) and mice exposed to 0 ppt PFOA + TMEV-infected (Group E; p < 0.0001). Both TMEV-infected groups (E and F) exhibited seizures at 14 dpi. However, the 70 ppt PFOA-exposed + TMEV-infected Group F mice had a significantly higher seizure frequency than mice exposed to 0 ppt PFOA + TMEV-infected (Group E; p < 0.01). Other common phenotypes observed in at least one mouse per group across the TMEV-infected groups, regardless of PFOA exposure (Groups E and F), included limb weakness and paralysis, backing up, and delayed righting reflex. Though these observations may be biologically relevant, not enough mice were affected to be considered statistically significant. Overall, mice exposed to both 70 ppt PFOA and TMEV infection displayed greater cumulative frequencies of phenotypic responses than those exposed to TMEV infection alone (**Figure 2B**). No sex-specific differences were found for any clinical signs except for retropulsion (p < 0.05) and delayed righting reflex response (p < 0.05) in mice exposed to 70 ppt PFOA + TMEV infection (Group F; **Figure 2A**).

### 3.4 Seizure activity in TMEV infected groups differed based on PFOA exposure

**Figure 3:**
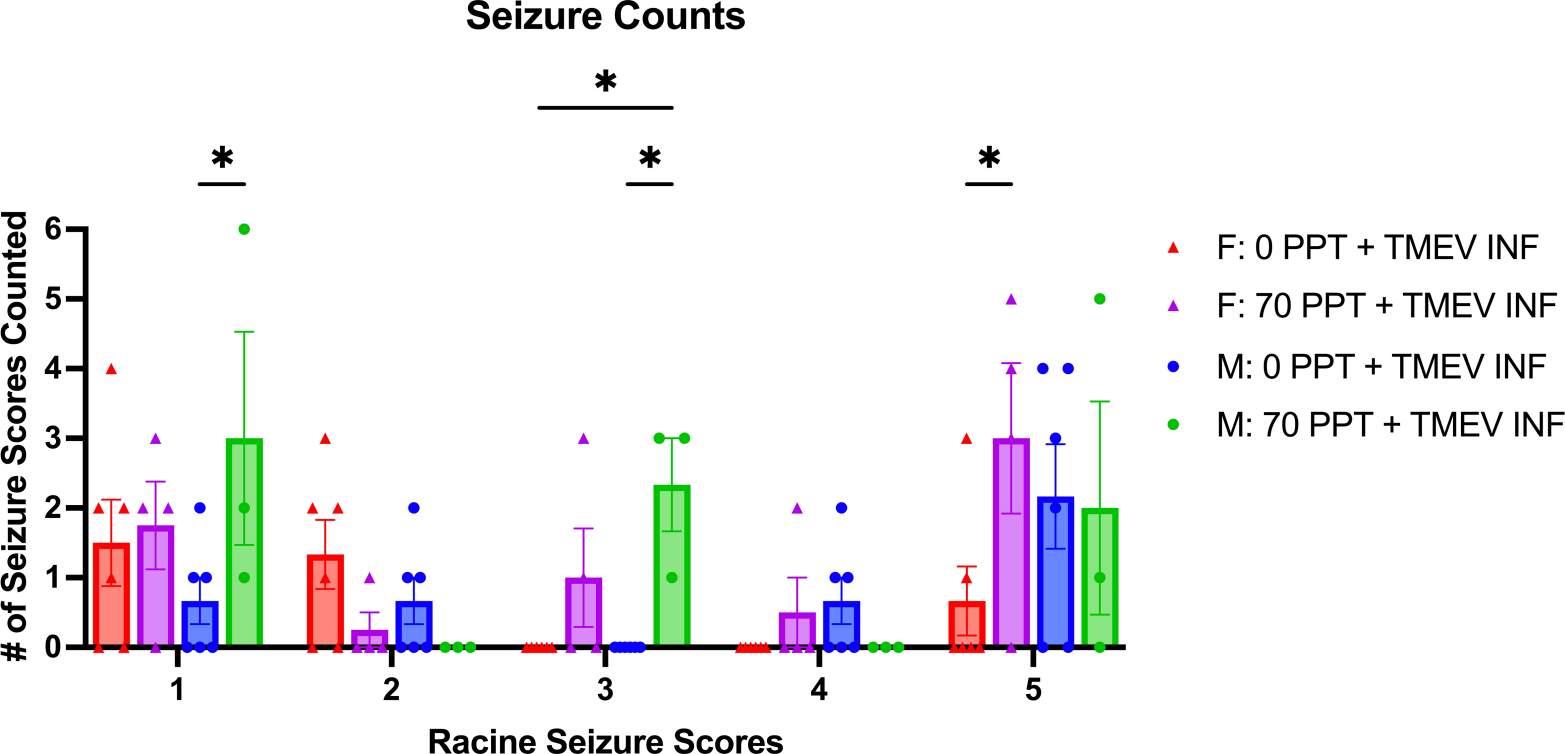
Mice were monitored for seizures twice a day for 14 days post-infection. The type of seizures was also tallied based on the Racine Seizure Score of 1–5. Seizure severities were compared across different treatment groups, indicated by different colors and shapes as shown in the figure legend. Females and males are shown separately, and each circular dot per score represents a mouse from Group E (0 ppt + TMEV infection) and each triangle per score from Group F (70 ppt + TMEV infection). *p < 0.05.

TMEV infection resulted in seizures; however, seizures were more predominant in TMEV-infected mice exposed to 70 ppt PFOA (Group F; **Figure 2A**). Seizures were observed in TMEV-infected mice not exposed to PFOA (Group E) between 4 and 7 dpi; whereas for TMEV-infected mice exposed to 70 ppt PFOA (Group F), seizures started as early as 2 dpi and ended at 8 dpi. The Racine seizure scale was used for clinical scoring purposes, with numerical scores given as follows: 1 - freezing with mouth and facial movement, 2 - freezing with head nodding, 3 - sitting with forelimb clasping, 4 - rearing with forelimb clasping, 5 - rearing and falling (**Figure 3**). Overall, seizures scoring 1, 3, and 5 were observed more often, with a score of 3 being significantly more common (p < 0.05) in TMEV-infected mice exposed to 70 ppt PFOA (Group F) versus 0 ppt PFOA (Group E). A score of 2 trended higher in TMEV-infected but unexposed mice (Group E), while scores of 4 were similarly noted in both TMEV-infected groups.

Sex-specific differences were also recorded in relation to the types of seizures observed in TMEV infected mice (**Figure 3**). Male mice exposed to 70 ppt PFOA and infected with TMEV (Group F) had significantly higher counts of seizures with scores 1 and 3 than TMEV-infected male mice with 0 ppt PFOA exposure (Group E; both p < 0.05). Furthermore, male mice exposed to 70 ppt PFOA and infected with TMEV (Group F) had more seizures with scores of 3 than TMEV-infected female mice with 0 ppt PFOA exposure (Group E; p < 0.05). In contrast, female mice exposed to 70 ppt PFOA and infected with TMEV (Group F) had more seizures with scores of 5 than TMEV-infected female mice with 0 ppt PFOA exposure (Group E; p < 0.05). Overall, seizures experienced by mice exposed to both PFOA and TMEV tended to have higher scores than those in mice exposed to TMEV alone.

### 3.5 TMEV RNA expression levels were not affected by 70 ppt PFOA exposure

**Figure 4:**
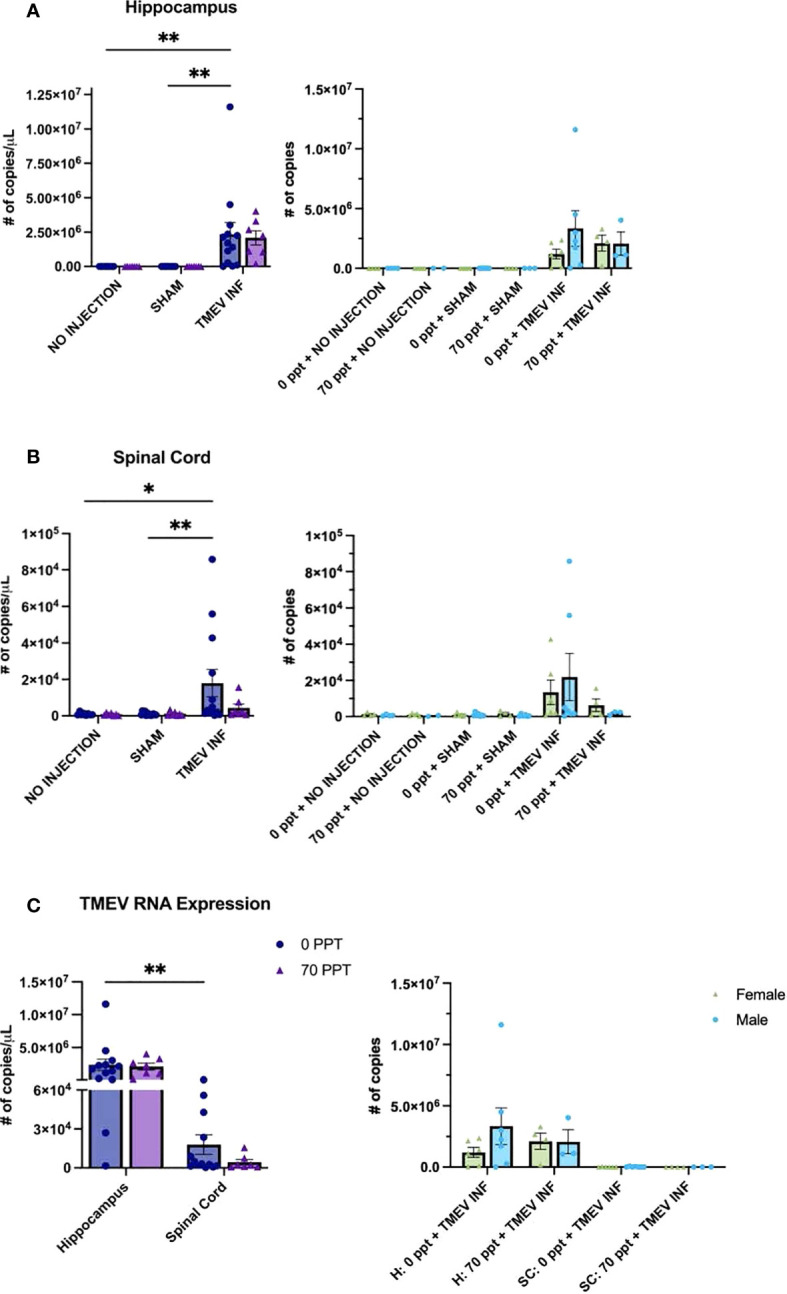
Real-Time quantitative PCR (RT-qPCR) was performed to measure TMEV RNA expression (number of transcript copies per µL) in CNS tissues at 14 dpi. Transcript levels were determined as follows: **(A)** Hippocampus, **(B)** Spinal cord, and **(C)** Comparison of both tissues of Groups E and F, both exposed to TMEV. Comparisons of values for females and males are shown to the right of each panel. For graphs on the left: • 0 ppt PFOA and Δ 70 ppt PFOA. For graphs on the right: females and males are distinguished by color and shape as shown in the figure legend in panel **(C)** *p < 0.05, **p < 0.01.

TMEV RNA expression was measured in the hippocampus and thoracic spinal cord, CNS tissues typically targeted by TMEV (47, 56, 57), for all mice infected with TMEV (**Figure 4**). Our interest in measuring TMEV RNA expression was to determine whether PFOA would suppress the immune system, resulting in higher viral RNA levels. In the hippocampus at 14 dpi (**Figure 4A**), TMEV-infected mice not exposed to PFOA (Group E) had, as expected, significantly higher levels of TMEV RNA than those of unexposed mice not injected or sham-infected (Groups A and C, both p < 0.01). While TMEV RNA was present in the hippocampus of TMEV-infected mice exposed to 70 ppt PFOA (Group F), differences in levels of TMEV RNA were not quite significant between not injected or sham-infected mice exposed to 70 ppt PFOA (Groups B and D, both p = 0.053). There were no significant differences in TMEV RNA expression in all TMEV-infected mice, regardless of PFOA exposure (Groups E and F), meaning both TMEV infected groups had similar levels of TMEV RNA, suggesting PFOA did not directly alter mechanisms of viral clearance.

Levels of TMEV RNA expression in the spinal cord resembled the expression found in the hippocampus (**Figure 4B**), as levels in TMEV-infected, unexposed mice (Group E) were significantly higher compared to those in the not injected and sham-infected unexposed mice (Groups A and C; p < 0.05 and p < 0.01). TMEV RNA expression was detected in the spinal cords of TMEV-infected mice exposed to 70 ppt PFOA (Group F), but expression levels did not significantly differ from those of the 70 ppt PFOA-exposed, not injected or sham-infected mice (Groups B and C). TMEV RNA transcript levels for TMEV-infected mice exposed to 70 ppt PFOA (Group F) were lower than those in TMEV-infected, unexposed mice (Group E), but did not significantly differ between the two groups. As expected, in both CNS tissues, the non-injected and sham-infected groups did not express TMEV RNA.

TMEV RNA expression was compared only in groups infected with TMEV (Groups E and F) between both tissues to determine the overall localization of TMEV RNA at this time point (**Figure 4C**). As expected, TMEV-infected, unexposed Group E had higher TMEV RNA expression in the hippocampus than in the spinal cord (p < 0.01). Although TMEV-infected, exposed to 70 ppt PFOA Group F tended to have lower TMEV RNA expression in the spinal cord, this was not significantly different from that found in the hippocampus (p = 0.084). No sex-related differences were identified in TMEV RNA expression between the different groups and tissues (**Figure 4**).

### 3.6 Cytokine and chemokine levels were altered after exposure to 70 ppt PFOA by PND 26

**Figure 5:**
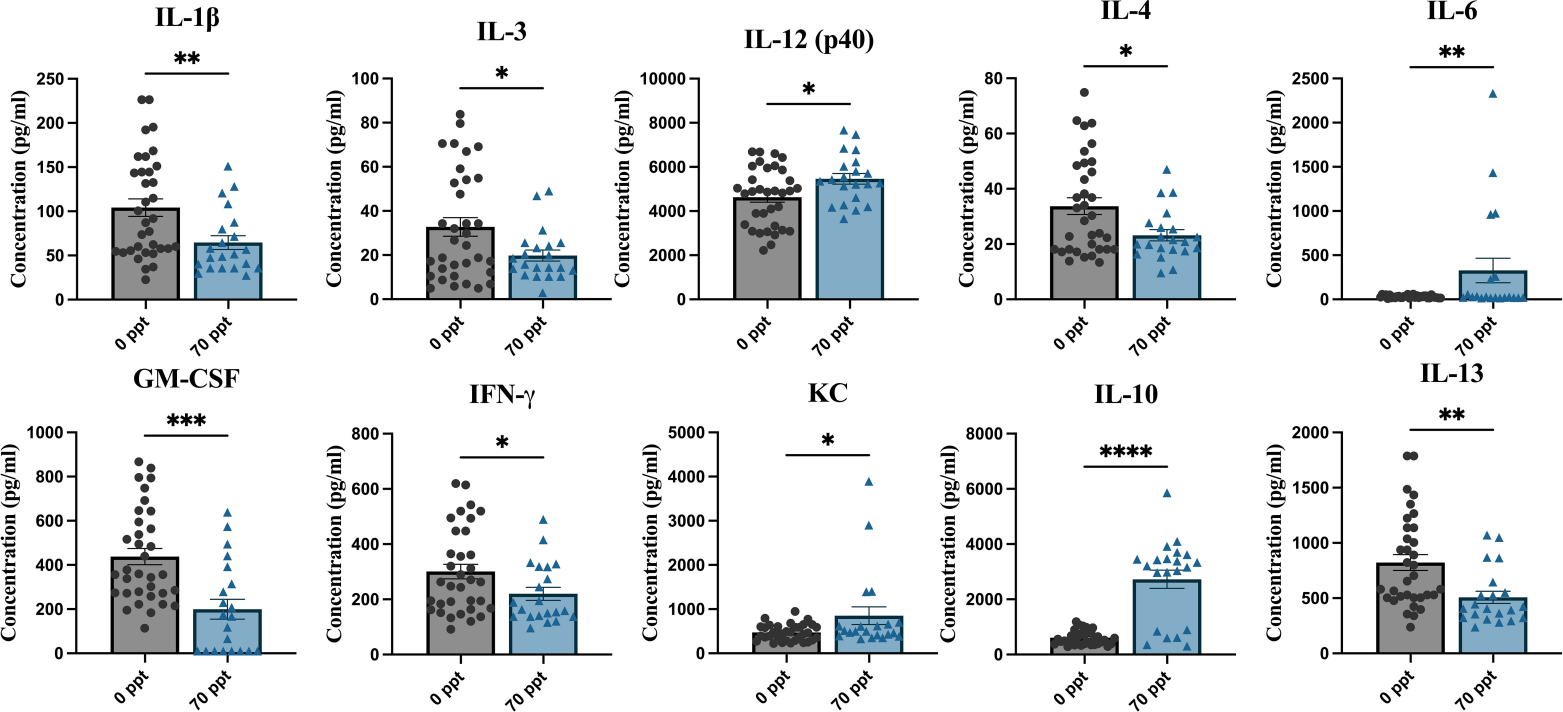
Pre-infection serum was collected at PND 25–26 to measure cytokine and chemokine levels. The samples were then separated by PFOA exposure. The different PFOA exposures can be found on the x-axis and the cytokine/chemokine concentration levels on the y-axis. *p < 0.05, **p < 0.01, ***p < 0.001, ****p < 0.0001.

Serum collected on PND 25–26, before i.c. injection, was used to measure baseline cytokine and chemokine levels. Prior to PND 28, mice were only exposed to either 0 ppt or 70 ppt PFOA. For this analysis, samples were grouped according to their PFOA exposure to evaluate the potential adverse effects of 70 ppt PFOA on the immune system by identifying any disruption in cytokine and chemokine levels (**Supplementary Figure 3**). Ten of the 23 cytokines investigated were significantly altered by exposure to 70 ppt PFOA (**Figure 5**). These cytokines included Th1 and cytokines IL-1β, IL-3, IL-12 [p40], GM-CSF, IFN-γ, and KC; Th2 and cytokines IL-4, IL-10, and IL-13; and IL-6, a cytokine that regulates cytokine/chemokine products of Th1 and Th2 cells. Interestingly, most of these cytokines and chemokines had lower levels in PFOA-exposed relative to non-PFOA exposed groups, as indicated by their low concentration levels compared to the control groups. PFOA-exposed mice had higher levels of IL-6, IL-10, IL-12 (p40), and KC compared to non-PFOA exposed mice. Overall, observations included a mixed response of Th1 cytokines and a suppression of Th2 cytokines, suggesting a PFOA-induced imbalance of Th1/Th2 cytokines.

Sex-related differences in cytokine and chemokine levels were examined between the two exposure groups (**Supplementary Figure 4**). Lower levels of IL-13 and GM-CSF were identified in males exposed to 70 ppt compared to their unexposed male counterparts (p < 0.05) and higher levels of IL-10 in both PFOA-exposed females and males compared to PFOA control females and males (p < 0.0001 and p < 0.001, respectively). Based on this study, the sex-specific disruptions may have originated from Th2 and Th17 cytokines.

### 3.7 Serum collected at PND 42 (14 dpi) revealed differences in cytokine and chemokine levels influenced by 70 ppt PFOA exposure

**Figure 6:**
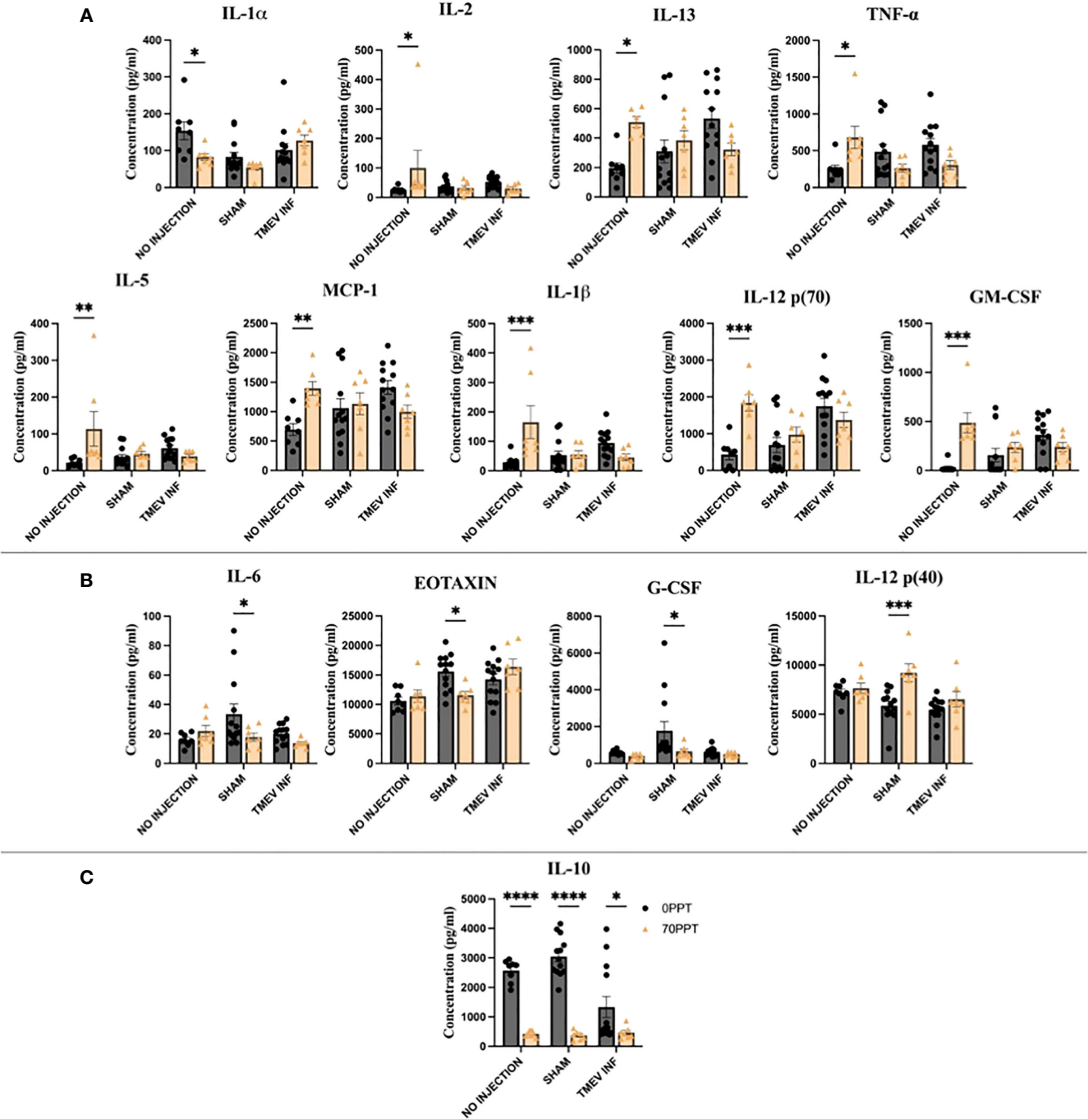
Serum collected on PND 42 (14 dpi) was used to compare cytokine and chemokine levels to identify PFOA-induced alterations. The cytokines highlighted above were arranged by the significance scores found for each TMEV-related group: **(A)** No infection, **(B)** Sham-infected, and **(C)** all three TMEV groups. • 0 ppt PFOA and Δ 70 ppt PFOA. *p < 0.05, **p < 0.01, ***p < 0.001, ****p < 0.0001.

Serum cytokine and chemokine levels at PND 42 (14 dpi for TMEV-infected groups) were measured to assess the longitudinal effects of PFOA exposure and determine whether PFOA altered immune responses after viral infection (**Supplementary Figure 5**). Cytokines and chemokines significantly affected by exposure to 70 ppt PFOA were identified by comparing the cytokine/chemokine levels of the 0 and 70 ppt PFOA groups in relation to TMEV infection status (non-infected, sham-infected, and TMEV-infected) (**Figure 6**).

The greatest disruption in cytokines and chemokines occurred in non-infected Groups A and B (**Figure 6A**). The cytokines IL-1β, IL-2, IL-12 [p70], GM-CSF, MCP-1, and TNF-α were produced at significantly higher levels than those in not-injected, 0 ppt PFOA Group A, apart from lower IL-1α levels from not-injected, 70 ppt PFOA exposure (Group B). Here, the cytokines IL-5 and IL-13 were produced at higher levels in the 70 ppt PFOA exposure, not injected, Group B. The findings among these two groups represent the sole effects of exposure to 42 consecutive days of 70 ppt PFOA, as these mice did not undergo the intracranial injection procedure.

In the analysis between sham-infected mice, four cytokines and chemokines significantly differed between 0 ppt and 70 ppt PFOA exposure groups (Groups C and D, **Figure 6B**). The chemokines Eotaxin and G-CSF were produced at lower levels in sham-infected mice exposed to 70 ppt PFOA (Group D). Similarly, IL-6 was produced at lower levels. In contrast, Th1-related IL-12 (p40) was produced at higher levels than in the sham-infected mice exposed to 0 ppt PFOA (Group C). The injection procedure itself can initiate an inflammatory response at 0 dpi (as previously reported (39)); whereas at 14 dpi PBS-injected (sham-infected) mice exposed to 0 or 70 ppt PFOA (Groups C and D) demonstrated differences in levels of cytokines and chemokines which were attributable to PFOA exposure.

Previously, immune responses after TMEV infection have been characterized throughout the acute phase (39). In this study, PFOA exposure and TMEV infection (Group F) resulted in low levels of IL-10 compared to mice exposed to 0 ppt PFOA + TMEV infection (Group E). Interestingly, IL-10 levels were low in all three TMEV exposure groups. Thus, subchronic exposure to PFOA resulted in suppressed IL-10 levels, regardless of viral infection.

Sex-specific differences were evaluated in cytokine and chemokine levels among each group (**Supplementary Figure 6**). Males exposed to 0 ppt PFOA + sham-infected (Group C) produced higher levels of IL-6 and G-CSF, and lower levels of RANTES, compared to females of Group C. Males also produced higher levels of MIP-1β than females when treated with 0 ppt PFOA + TMEV infection (Group E).

### 3.8 PFOA-induced cytokine alterations influenced TMEV RNA levels and seizure frequency

A Pearson R correlation analysis was performed to determine whether cytokine and chemokine levels in mice infected with TMEV and exposed to either 0 ppt or 70 ppt PFOA (Groups E and F) were associated with hippocampal and spinal cord TMEV RNA expression levels (**Supplementary Figure 7**). As expected, no direct correlations were found. A similar analysis was performed using the cytokine and chemokine levels from both groups to assess their influences on the 14 dpi seizure frequency scores (**Supplementary Figure 8**). Similarly, the association strength of cytokine and chemokine levels with seizure scores was low for TMEV-infected mice exposed to 0 ppt PFOA (Group E; r = – 0.37 to 0.46). In contrast, seizure frequency scores were associated with cytokine and chemokine levels for TMEV-infected mice exposed to 70 ppt PFOA (Group F): five cytokine levels with low associations (r = – 0.22 to 0.10), six cytokine levels with moderate associations (r = – 0.69 to – 0.53, 0.61), and 12 cytokines with strong negative associations (r = – 0.80 to – 0.71). Of these, IL-12 (p70) had the strongest correlation (r = – 0.80, p < 0.05) with seizure frequency (**Table 2**). IL-12 (p70) stimulates the growth and function of T cells, production of TNF-α from T cells and NK cells, and IFN-γ by reducing IL-4 mediated suppression of IFN-γ, thus promoting an inflammatory environment contributing to the onset of seizures (58).

**Table 2:** (A) Pearson R correlation analysis was performed to find associations between cytokines and chemokines of Group F mice (PFOA + TMEV) and 14 dpi seizure frequency scores.

Cytokines	IL-1β	IL-6	IL-9	IL-12 (p70)	IL-17α	IFN-γ	MCP-1
R score	-0.77	-0.78	-0.78	-0.80	-0.76	-0.77	-0.79
P-value	<0.05	<0.05	<0.05	<0.05	<0.05	<0.05	<0.05

P values not shown were above 0.05.

## 4 Discussion

Early life exposure to perfluorinated compounds, such as PFOA, has been linked to disruptions of the immune system, debilitating the line of defense crucial to resisting viral and environmental stressors. Although the exact molecular network remains unclear, several studies have indicated a potential role of PFOA in suppressing immune responses, e.g., reduction of antibody response levels, alteration of T cell proliferation, and activation of peroxisome proliferator activated receptor (PPAR)-α and γ (59, 60). Exposure to PFOA during brain development has been associated with changes in spontaneous behavior, susceptibility of the cholinergic system, alteration of axonal and dendritic cell outgrowth, synaptogenesis, and proliferation of glial cells, thus increasing susceptibility to neurological conditions (61). Typically, environmental pollutant exposures occur at low doses for a chronic duration, which differs from most of the existing high-dose and short-term *in vivo* and *in vitro* studies. In this study, the adverse effects of PFOA were evaluated using a dose and route of administration relevant to human exposures of PFOA, following EPA’s drinking water standard (70 ppt) and using an established mouse model of neurotropic viral infections in humans. Exposure *via* breast milk and drinking water have both been associated with adverse effects in humans and mice, and while breast milk tends to have higher concentrations of PFAS including PFOA, it is unclear whether PFOA levels in drinking water are substantially lower (3, 43, 62).

Not only do chemical compounds have the capacity to compromise the immune system, but when susceptible individuals also encounter biological health hazards (e.g., bacteria, parasites, and viruses), they may not proficiently eliminate the encountered pathogen. Historically, the TMEV model has been used to study viral-induced neurological diseases such as ALS, epilepsy, MS, and PD. The responses to TMEV infection vary by mouse strain, producing distinct clinical symptoms and cytokine/chemokine profiles which vary in effectiveness at eliminating viral replication (39, 45). For this study, the TMEV-resistant B6 strain was chosen for its well-known ability to clear the virus despite experiencing viral-induced seizures influenced by a low Th2 cytokine production (57, 63). Based on the known adverse effects of PFOA on the immune system (i.e., rendering an imbalance between Th1 and Th2 cytokine production (20, 64)), exposure to 70 ppt PFOA was hypothesized to suppress immune responses responsible for viral clearance and the worsening of viral-induced neurological symptoms.

After exposure to a low dose of 70 ppt PFOA in drinking water for 26 and 42 days, PFOA was bioavailable and accumulated in the serum (**Supplementary Figure 1**). The PFOA levels detected at the end of the 42-day experiment were approximately half of the levels after 26 days, correlating with the serum half-life of PFOA in the literature. Exposure to PFOA resulted in increased weight ratios compared to control mice and to mice infected with TMEV. The results agree with previous research that found that exposure to PFOA resulted in maternal weight gain (65–67) and obesity in children (68–70). While PFOA-adiposity mechanisms remain unclear, potential pathways have been proposed (e.g., PPAR-α receptors increase lipid metabolism and adipocyte differentiation (8), decreasing thyroxine and increasing thyroid stimulating hormone (71, 72), and decrease of circulating estrogen concentrations (73, 74)).

During brain development and maturation, environmental toxins can modify brain hormone systems and increase susceptibility to neurological conditions (75, 76). PFAS can cross the blood brain barrier to exert effects on CNS homeostasis (22, 61, 77, 78). Early-life exposures to legacy PFAS have been associated with suppressed immune responses, altered cholesterol levels, and abnormal growth and development (10, 12, 19, 79). Acute and chronic PFAS exposure has also been shown to interfere with motor function, myelination, microglial activation, and pro-inflammatory cytokine transcript expression (13, 61, 80, 81). However, few studies have assessed the effects of PFOA exposure on circulating cytokines and chemokines.

Cytokines and chemokines are essential for cell signaling during the immune response. A notable immunotoxic effect has been shown to be induced by PFOA on T-cell dependent IgM antibody, responsible for host defense against infections (14, 82). T helper cells (Th cells) are of particular interest since Th cell differentiation modulates the direction and magnitude of an immune response by their cytokine and chemokine production. A 26-day ad libitum exposure to 70 ppt PFOA resulted in a mixed response for systemic Th1 and Th2 cytokines, but overall favoring the suppression of Th2 cytokines (IL-4 and IL-13). High levels of cytokines IL-6 and IL-10 were identified, but these cytokines may be produced from other lymphocyte cells and T regulatory cells (83, 84). Our results coincide with another study in which B6C3F1 mice were orally exposed to low and high doses of PFOA, suggesting PFOA induced an imbalance between Th1 and Th2 cytokines (82). However, currently there are few other studies supporting these observations. While other high-dosed exposure studies agree that PFOA was associated with a shift between Th1 and Th2 cytokines, these studies instead suggest a suppression of Th1 cytokines (64, 85). The mixed results in current literature supporting whether PFOA polarizes Th1 or Th2 cytokines highlights a need for future PFOA exposure studies, because T-regulatory cells may have a greater role to prevent any pathogenic effects of the immune response.

Cytokine and chemokine levels were measured at PND 42 to evaluate the effects of PFOA coincident to the acute phase of infection (14 dpi). Surprisingly, the non-injected groups A and B had, between them, the most altered cytokine and chemokine levels. Since both groups did not undergo an intracranial injection, the differences in levels were interpreted as the result of the 42-day ad libitum exposure to PFOA. Compared to the Th2 cytokine suppression found at PND 26, the responses at PND 42 indicated a shift toward a balanced Th1 and Th2 environment. Apart from low IL-1α levels, both pro- and anti-inflammatory cytokine levels were higher in the PFOA exposed mice. Within the elevated Th1 cytokines, the levels of IL-1β and TNF-α were notable as these belong to the main inflammatory triad, responsible for a mediated host response and resistance to pathogens. However, uncontrolled levels may result in exacerbated damage during chronic disease and acute tissue injury (86–88). The increase in inflammatory levels have been observed in other PFOA exposure studies; however, most of those levels were characterized based on gene expression in several organs and cells. For instance, a 21-day exposure to 200 ppm PFOA in drinking water resulted in an increased expression of the same pro-inflammatory cytokines, IL-1β and TNF-α, in the spleen (89). Another reported increased levels of both cytokines in mast cells treated with a PFOA exposure (50 – 400uM) of 12 hours (90). Overall, the increased Th1 cytokine levels suggested an inflammatory response in attempt to manage systemic PFOA levels, while the Th2 cytokines served as mediators for a controlled inflammatory environment.

Cytokine levels between Groups C and D were compared to determine the influence of PFOA on sham-infected mice at 14 dpi. Most cytokines and chemokines returned to basal levels, with the exception of high levels of IL-12 (p40) and low levels of G-CSF, IL-6, and Eotaxin. These two groups underwent the i.c. injection procedure, which has been previously associated with the release of cytokines and chemokines, to address the effect of this injury (39). A strong suppression of cytokine levels was expected due to both PFOA and TMEV exposure (Group F), but in fact only levels of IL-10 were lower compared to the PFOA-control counterpart. Interestingly, low IL-10 levels were observed in the no-injection, sham-injection, and TMEV infection groups also exposed to PFOA. Therefore, the alterations observed were influenced specifically by PFOA exposure. The association between PFOA and cytokine profiles has been described in Chinese women of childbearing age, indicating a negative association with IL-10 (91). PFOA also decreased IL-10 in phytohaemagglutinin (PHA)-stimulated peripheral blood leukocytes (17). In addition, decreased IL-10 expression has been noted in B6 mice after exposure to PFOA (0 – 3 mg/kg for 35 days) (23). IL-10 deficiency may enhance pathogenic clearance during an acute infection, but may also exaggerate the inflammatory response, leading to tissue damage (92–94). Thus, IL-10 is likely critical in disease processes affected by PFOA exposure, due to its therapeutic potential and resolution of host inflammatory responses.

Currently, therapeutic strategies for immunological disorders focus on skewing a balanced Th1/Th2 production (95–97). Susceptibility to virus-induced neurological symptoms has also been characterized by an increased shift in Th2 cytokines, resulting in chronic infections due to a strong anti-inflammatory environment. There is evidence of polarization towards the Th2 immune phenotype in immunological pathologies such as asthma and systemic lupus erythematosus (98). A high response of Th2 cytokines have been associated with severe stages in COVID-19 patients, rather than a Th1 response (99). Thus, high stimulation of Th1 cytokines, for instance in B6 mice infected with TMEV, has been shown to clear virus infection due to an active inflammatory response that allows for proper viral clearance (100). In addition, this robust inflammatory response results in the induction of seizures during the first week post-infection; however, these seizures largely resolve by the end of the acute phase, although epilepsy develops later once the levels of inflammation return to normal (101). The influence of cytokines on seizures have been characterized by the increased expression of the cytokines IL-1β, IL-6, TNF-α, and IFN-γ (102). In the current study, we observed a higher frequency of seizures in Group F mice (70 ppt PFOA + TMEV). However, there were no differences in cytokine levels in this group at PND 42 (end of the acute phase), except for low levels of IL-10, contrary to expectations. Serum was collected at 14 dpi, and seizures had already ceased by 8 dpi; thus, cytokines and chemokines had returned to basal levels by 14 dpi.

TMEV RNA has been measured previously in the hippocampus and spinal cord of infected mice (47, 56), and epileptic seizures following TMEV infection of C57BL/6 mice have been associated with pathological changes to the hippocampus (103). To further determine whether the PFOA-induced immune response affected viral clearance, TMEV RNA expression was measured in the hippocampus and spinal cord of TMEV-exposed groups. However, viral RNA expression levels were similar in both the control and PFOA-exposed groups (Group E and F). Thus, PFOA-induced alterations in the cytokine response, characterized by decreased IL-10 levels, did not influence the mechanisms underlying viral clearance. Similarly, mice deficient in IL-10 were able to resolve viral loads of Lymphocytic Choriomeningitis Virus (LCMV) in uterine tissue at earlier rates than in wild-type mice (104). However, prolonged IL-10 deficiency may also be pathogenic. For instance, IL-10 knockout mice infected with mouse hepatitis virus exhibited morbidity without affecting viral clearance (105).

Pearson’s correlation revealed relationships between cytokine and chemokine levels at PND 42 and seizure frequency scores and TMEV RNA expression. No correlations were found between cytokine levels and TMEV RNA expression; however, cytokines IL-1β, IL-9, IL-12 (p70), IL-17, IFN-γ, MCP-1, and IL-6 had a significant inverse correlation with 14 dpi seizure frequency scores. Though an inverse relationship was identified between these pleiotropic cytokines and seizure frequency, an inflammatory environment typically induces the onset of seizures (58).

Overall, our findings demonstrated the effects PFOA exposure had on viral-induced phenotypes and immune responses. This study is one of the first to determine the systemic cytokine and chemokine levels affected by PFOA at low concentrations. These results suggest a protective role of PFOA exposure in viral-induced neurological disease, as previous studies have shown that a shift towards a high Th1 cytokine production results in viral clearance (100). One of our limitations is the diversity in the produced cytokine and chemokine levels between the different exposure groups. Thus, future studies analyzing the temporal changes throughout the polarization between Th1 and Th2 cytokines are pertinent to determine when an imbalance occurs and begins to stimulate pathogenic effects. Another limitation was the PFOA concentration found in the serum of both non-injected Group A and B. PFAS are expected to be found in the control water due to their functional groups, carbon chain length, hydrophilicity, and hydrophobicity properties (106). However, filtered water consumption can reduce PFOA concentration in serum compared to regular unfiltered water (107). While the concentration levels did not significantly differ between Groups A and B in the serum, future investigations using the brain, liver, and kidneys to quantify for PFOA levels would indicate the chemical fate and place of bioaccumulation to understand the areas at risk for influencing a multifaceted biological response. While subtle alterations in serum cytokine and chemokine levels may have contributed to an increased frequency of viral-induced seizures, PFAS could have accelerated symptom onset or exacerbated symptom severity by causing damage to the blood brain barrier (BBB) (77). The severity of neurological symptoms (e.g., MS and AD) have been shown to be tightly coupled with BBB integrity (108). Thus, heightened damage by PFOA exposure could compromise the BBB and render the host susceptible to potential pathogens. Future studies are needed to determine whether an increase in seizures was caused by PFOA-induced BBB permeability. Additional efforts should also be made to determine whether this increased permeability affects TMEV dispersion in the CNS outside of the hippocampus and spinal cord. These results are of the few to highlight the cytokine and chemokine alterations affected by a low dose of PFOA; however, continuation of these studies at a variety of doses and mixtures of PFAS are necessary to fully evaluate the implications of PFAS and to further support regulatory measurements.

## Data availability statement

The original contributions presented in the study are included in the article/**Supplementary Material.** Further inquiries can be directed to the corresponding author.

## Ethics statement

The animal study was reviewed and approved by Texas A&M University Laboratory Animal Care and Use Committee.

## Author contributions

Conceptualization, CB-L, CW, and TP; validation and formal analysis, AP; investigation, AP, MW, KK, KA and CY; resources, CB-L, CW and TP; data curation, CB-L, AP; writing—original draft preparation, AP; writing—review and editing, MW, CB-L and CW; visualization, AP; supervision, CB-L; project administration, CB-L; funding acquisition, CB-L. All authors contributed to the article and approved the submitted version.
