# Supplementary material for: C57BL/6J mice exposed to perfluorooctanoic acid demonstrate altered immune responses and increased seizures after Theiler’s murine encephalomyelitis virus infection
Source: Front Immunol. 2023 Aug 2;14:1228509. doi: 10.3389/fimmu.2023.1228509 (PMC10434537; doi:10.3389/fimmu.2023.1228509)
Supplement: Supplementary file 1 [file DataSheet_1.docx]

Supplementary Material

**C57BL/6J mice exposed to perfluorooctanoic acid demonstrate altered immune responses and increased seizures after Theiler’s murine encephalomyelitis virus infection**

**Aracely A. Pérez Gómez, Meichen Wang, Kelli Kochan, Katia Amstalden, Colin R. Young, C. Jane Welsh, Timothy Phillips, and Candice Brinkmeyer-Langford***

*** Correspondence:** brinkmeyer@tamu.edu

# Supplementary Figures and Tables

| Tukey's multiple comparisons test | Predicted (LS) mean diff. | 95.00% CI of diff. | Below threshold? | Summary | Adjusted P Value |
| --- | --- | --- | --- | --- | --- |
| 0PPT + SHAM vs. 0PPT + NO INFECTION | 0.0684 | -0.05969 to 0.1966 | No | ns | 0.6474 |
| 0PPT + SHAM vs. 0PPT + TMEV INF | -0.0549 | -0.1667 to 0.05693 | No | ns | 0.7253 |
| 0PPT + SHAM vs. 70PPT PFOA + NO INFECTION | -0.2096 | -0.3503 to  -0.06888 | Yes | *** | 0.0003 |
| 0PPT + SHAM vs. 70PPT + SHAM | -0.2396 | -0.3733 to  -0.1060 | Yes | **** | <0.0001 |
| 0PPT + SHAM vs. 70PPT + TMEV INF | -0.0749 | -0.2085 to 0.05881 | No | ns | 0.5986 |
| 0PPT + NO INFECTION vs. 0PPT + TMEV INF | -0.1233 | -0.2515 to 0.004782 | No | ns | 0.0669 |
| 0PPT + NO INFECTION vs. 70PPT PFOA + NO INFECTION | -0.2780 | -0.4320 to  -0.1241 | Yes | **** | <0.0001 |
| 0PPT + NO INFECTION vs. 70PPT + SHAM | -0.3081 | -0.4556 to  -0.1605 | Yes | **** | <0.0001 |
| 0PPT + NO INFECTION vs. 70PPT + TMEV INF | -0.1433 | -0.2909 to 0.004271 | No | ns | 0.0627 |
| 0PPT + TMEV INF vs. 70PPT PFOA + NO INFECTION | -0.1547 | -0.2954 to  -0.01398 | Yes | * | 0.0216 |
| 0PPT + TMEV INF vs. 70PPT + SHAM | -0.1847 | -0.3184 to  -0.05106 | Yes | ** | 0.0012 |
| 0PPT + TMEV INF vs. 70PPT + TMEV INF | -0.0200 | -0.1536 to 0.1137 | No | ns | 0.9982 |
| 70PPT PFOA + NO INFECTION vs. 70PPT + SHAM | -0.0300 | -0.1887 to 0.1286 | No | ns | 0.9945 |
| 70PPT PFOA + NO INFECTION vs. 70PPT + TMEV INF | 0.1347 | -0.02388 to 0.2934 | No | ns | 0.1482 |
| 70PPT + SHAM vs. 70PPT + TMEV INF | 0.1648 | 0.01236 to 0.3172 | Yes | * | 0.0253 |

**Supplementary Table 1.** Mouse weights taken at PND 42 (14 days post-infection) were compared via two-way ANOVA. The leftmost column lists treatment groups compared; the column labeled “Predicted (LS) mean diff.” gives the difference between predicted least squares means values for the compared groups. The column labeled “95.00% CI of diff.” shows the lower and upper bounds of the 95% confidence interval for these differences. “Below threshold?” indicates which comparisons met the criteria for being below the threshold set to define significance, and “Summary” shows which comparisons were not significant (ns), or significant (* p < 0.05, ** p < 0.01, *** p < 0.001, **** p < 0.0001). Adjusted p-values are listed in the rightmost column.


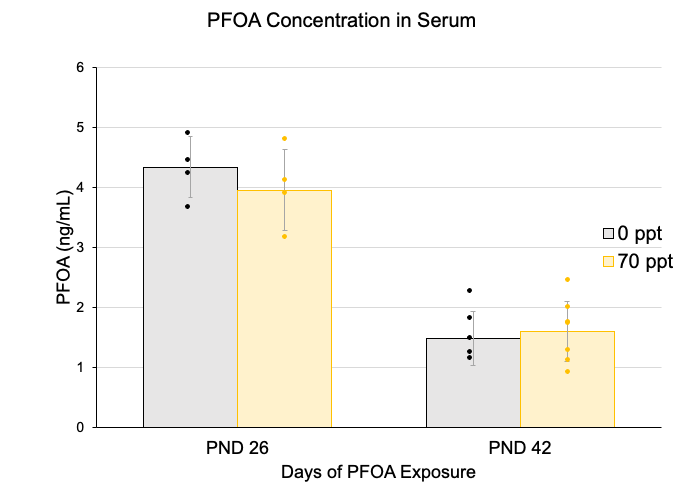


**Supplementary Figure 1**. Serum collected at postnatal day (PND) 26 (before infection) and at PND 42 (end of study) was used to measure PFOA concentration levels via mass spectrometry. The presence of PFOA in the 0 ppt sample reflects baseline PFOA contamination in water.


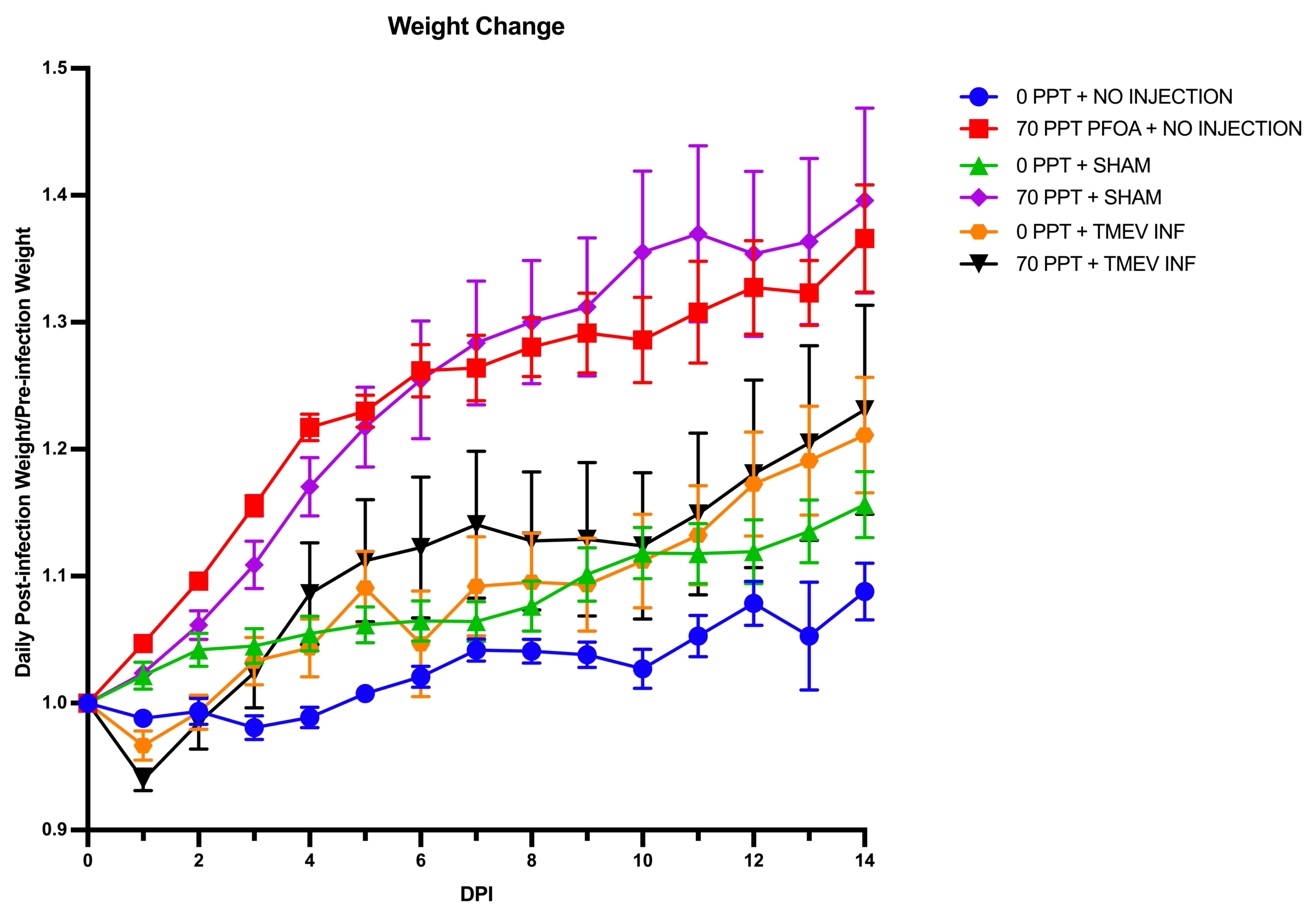


**B)**

**A)**

**Supplementary Figure 2**. Weights were measured for each day from 0-14 dpi, and normalized against 0dpi weights to show actual weight gained or lost. A) Weight ratio changes were compared between the exposure groups. Ratios were calculated by dividing the weight measured at a specific time point by the initial weight before any i.c. injection. B) The effects of sex are also shown with different colors and shapes. Treatment groups are indicated by the title of each graph. Error bars indicate standard error of mean (s.e.m.).

**Supplementary Figure 3**. Cytokine and chemokine levels were measured and compared between mice exposed to 0 ppt or 70 ppt PFOA. Treatment is shown on the x-axis, and cytokine/chemokine concentrations are shown on the y-axis. * p < 0.05, ** p < 0.01, *** p < 0.001, **** p < 0.0001

**Supplementary Figure 4**. Cytokine and chemokine levels were measured and compared between female and male mice exposed to 0 ppt or 70 ppt PFOA. Sex is shown on the x-axis, and cytokine/chemokine concentrations are shown on the y-axis. Treatment is indicated by color and shape, as shown in the legend. * p < 0.05, ** p < 0.01, *** p < 0.001, **** p < 0.0001

**Supplementary Figure 5**. Serum cytokine and chemokine levels at PND 42 (14 dpi for TMEV-infected groups) were measured to determine whether PFOA altered immune responses after viral infection. Infection status is shown on the x-axis and cytokine/chemokine concentrations are shown on the y-axis. PFOA exposure is indicated by color and shape, as shown in the legend.

* p < 0.05, ** p < 0.01, *** p < 0.001, **** p < 0.0001

**Supplementary Figure 6**. Cytokine and chemokine levels were measured and compared between female and male mice from different treatment groups. Treatment groups are shown along the x-axis, and cytokine/chemokine concentrations are shown on the y-axis. Sex is indicated by color and shape, as shown in the legend.

* p < 0.05, ** p < 0.01

**Supplementary Figure 7**. Pearson R correlation analyses revealed strength of associations between cytokine, chemokine, or TMEV RNA expression measured in the hippocampus or spinal cord, for TMEV-infected mice from Groups E (top) and F (bottom).


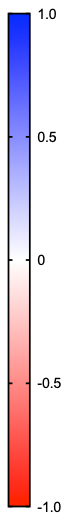

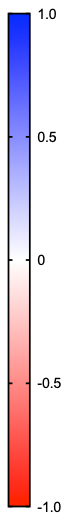


**Supplementary Figure 8**. Pearson R correlation analyses revealed strength of associations between cytokine, chemokine, or frequencies of seizures for TMEV-infected mice from Groups E (top) and F (bottom).
